# NVP-BEZ235 and NVP-BGT226, dual phosphatidylinositol 3-kinase/mammalian target of rapamycin inhibitors, enhance tumor and endothelial cell radiosensitivity

**DOI:** 10.1186/1748-717X-7-48

**Published:** 2012-03-27

**Authors:** Emmanouil Fokas, Michio Yoshimura, Remko Prevo, Geoff Higgins, Wolfgang Hackl, Sauveur-Michel Maira, Eric J Bernhard, W Gillies McKenna, Ruth J Muschel

**Affiliations:** 1 Gray Institute for Radiation Oncology and Biology, Oxford University, Oxford, UK; 2 Novartis Pharma AG, Novartis Campus, CH-4057 Basel, Switzerland; 3 Gray Institute of Radiation Oncology and Biology, University of Oxford, Oxford OX3 7DQ, UK

**Keywords:** PI3K, mTOR, Radiosensitization, Endothelial cells, VEGF

## Abstract

**Background:**

The phosphatidylinositol 3-kinase (PI3K)/Akt pathway is activated in tumor cells and promotes tumor cell survival after radiation-induced DNA damage. Because the pathway may not be completely inhibited after blockade of PI3K itself, due to feedback through mammalian target of rapamycin (mTOR), more effective inhibition might be expected by targeting both PI3K and mTOR inhibition.

**Materials and methods:**

We investigated the effect of two dual PI3K/mTOR (both mTORC1 and mTORC2) inhibitors, NVP-BEZ235 and NVP-BGT226, on SQ20B laryngeal and FaDu hypopharyngeal cancer cells characterised by EGFR overexpression, on T24 bladder tumor cell lines with H-Ras mutation and on endothelial cells. Analysis of target protein phosphorylation, clonogenic survival, number of residual γH2AX foci, cell cycle and apoptosis after radiation was performed in both tumor and endothelial cells. In vitro angiogenesis assays were conducted as well.

**Results:**

Both compounds effectively inhibited phosphorylation of Akt, mTOR and S6 target proteins and reduced clonogenic survival in irradiated tumor cells. Persistence of DNA damage, as evidenced by increased number of γH2AX foci, was detected after irradiation in the presence of PI3K/mTOR inhibition, together with enhanced G2 cell cycle delay. Treatment with one of the inhibitors, NVP-BEZ235, also resulted in decreased clonogenicity after irradiation of tumor cells under hypoxic conditions. In addition, NVP-BEZ235 blocked VEGF- and IR-induced Akt phosphorylation and increased radiation killing in human umbilical venous endothelial cells (HUVEC) and human dermal microvascular dermal cells (HDMVC). NVP-BEZ235 inhibited VEGF-induced cell migration and capillary tube formation in vitro and enhanced the antivascular effect of irradiation. Treatment with NVP-BEZ235 moderately increased apoptosis in SQ20B and HUVEC cells but not in FaDu cells, and increased necrosis in both tumor and endothelial all cells tumor.

**Conclusions:**

The results of this study demonstrate that PI3K/mTOR inhibitors can enhance radiation-induced killing in tumor and endothelial cells and may be of benefit when combined with radiotherapy.

## Background

Radiotherapy is one of the most important modalities for the management of cancer. However, despite progress in radiation technology and significant gains achieved with the use of combined radio-chemotherapy, there is a substantial proportion of patients that fail to achieve long-term control [1]. The latter provides a strong rationale for combining molecular targets with radiation to improve patient outcome.

The phosphatidylinositol 3-kinase (PI3K)/Akt/mammalian target of rapamycin (mTOR) pathway controls tumor cell proliferation, growth, and survival after DNA damage [2]. Activation of this pathway is frequent in many cancers and can occur through diverse mechanisms such as amplification of the epidermal growth factor receptor (EGFR) gene, mutations of the Ras oncogene, PI3K mutations and loss of phosphatase and tensin homologue deleted in chromosome 10 (PTEN) [1-3]. This pathway consists of EGFR/Ras/PI3K/Akt and is a prime target for inhibition in the context of radiotherapy [4-6]. We and others have previously shown that inhibition of the EGFR/Ras/PI3K/Akt pathway can increase susceptibility to radiation-induced tumor killing [3,7-11]. Inhibition of Ras, PI3 kinase and Akt reduce tumor clonogenic survival after radiation at clinically relevant doses [3-5,7,10,12]. A phase III randomized clinical trial evaluated the addition of cetuximab, an EGFR inhibitor, to radiotherapy and demonstrated improved overall survival in the combined modality arm over radiation alone [13].

The kinase mTOR consists of TORC1 and TORC2, two functionally distinct multiprotein complexes [14]. TORC1 includes mTOR and raptor (regulatory-associated protein of mTOR). TORC2 is composed of mTOR and rictor (rapamycin-insensitive companion of TOR) and regulates the activity of Akt [14]. mTOR inhibitors have radiosensitising potential in tumor and vascular cells [15,16]. Inhibition of TORC1 activity alone can result in TORC2-mediated feedback phosphorylation of Akt on Ser473 [14,17]. The paradoxical feedback activation of the PI3K/Akt pathway may compromise the efficacy of TORC1 inhibitors and provide the rationale for generating dual inhibitors.

Preclinical studies have demonstrated antitumor activity for the PI3K/mTOR inhibitor NVP-BEZ235 (BEZ235) in a variety of models especially those with PI3K mutation or K-Ras mutation [18,19]. Here, we examined whether the PI3K/mTOR (both mTORC1 and mTORC2) inhibitors BEZ235 and NVP-BGT226 (BGT226) could sensitise tumor cells with EGFR overexpression or Ras mutation to radiation. We investigated two inhibitors to get a better insight of the efficacy of each compound and test whether comparable results will be obtained. Both dual PI3K/mTOR inhibitors are issued from the same chemical space (Imidazo-quinolines). BGT226 displays more prolonged effects on target in cells, likely due the slow kinetics on target (high affinity, slow release). Additionally, we studied how PI3K/mTOR inhibition can modify the response of endothelial cells after IR. A substantial body of evidence has demonstrated that the PI3K/mTOR pathway is involved in angiogenesis and functions downstream of vascular endothelial growth factor (VEGF) to promote endothelial cell survival [20-22]. We therefore tested the impact of one the inhibitors, BEZ235, on VEGF-mediated Akt signaling, survival and in vitro angiogenesis in irradiated tumor and endothelial cells.

## Methods

### Cell culture

T24 bladder and FaDu hypopharyngeal cancer cell lines were obtained from ATCC. SQ20B laryngeal squamous cell carcinoma cells were obtained from Dr. Ralph Weichselbaum (University of Chicago, Chicago, IL). Tumor cells were cultured as described [7]. Human umbilical vein endothelial cells (HUVEC) and human dermal microvascular cells (HDMVC) were purchased from Lonza and were maintained in EGM-2 medium (Lonza) supplemented with EGM-2 SingleQuots (Lonza) at 37°C in water saturated 5% CO2/95% air.

### Dual PI3K/mTOR inhibitors treatment

BGT226 and BEZ235 dual PI3K/mTOR inhibitors were obtained from Novartis Pharma AG. The drugs were added to mid-log phase cell cultures. After treatment, medium was replaced with drug-free medium. For the control group, equal amounts of DMSO were used.

### Clonogenic survival assay

The effect of BEZ235 (50 nmol/L) and BGT226 (5 nmol/L) on tumor cell survival after irradiation was assessed by clonogenic assay, as previously reported [7]. Different drug-radiation schedules were tested (see "Results"). In HUVEC and HDMVC, BEZ235 (50 nmol/L) was added 1 h before radiation and medium was replaced by basal medium containing 1.5% FCS and a constant concentration of VEGF (10 ng/ml) at 1 h post-irradiation [23].

We also assessed clonogenicity in tumor cells cultured in hypoxia after treatment with one of the PI3K/mTOR inhibitors, BEZ235 (50 nmol/L). For the clonogenic assays performed in hypoxia, tumor cells were incubated in 0.5% O2 using an InVivo_2 _300 chamber (Ruskinn Technology, UK), for 6 h before irradiation under hypoxic conditions using tightly sealed chambers. The target O_2 _level was achieved within 6 h of gassing and maintained during irradiation, as confirmed by an OxyLite oxygen probe (Oxford Optronix). Tumor cells irradiated under hypoxia were exposed to normoxia at 1 h post-irradiation. As standard, BEZ235 was added 1 h prior to irradiation and was washed away 17 h after irradiation.

### Analysis of protein phosphorylation

Immunoblotting was performed as described elsewhere [7]. Blocking was performed by 5% bovine serum albumin for phospho-specific antibodies. Phospho-mTOR (ser-2448), phospho-Akt (ser-473) and phospho-S6 (ser-235/236) primary antibodies (Cell Signaling) were used at 1:1,000 dilution. β-actin clone AC-15 (Sigma) was used at 1:4,000 dilution. Antibody binding was detected with enhanced chemiluminescence kit (GE-Amersham).

### Analysis of γH2AX foci

Residual DNA damage in irradiated FaDu and SQ20B cells was assessed by measuring residual γH2AX foci. Cells were pretreated with either BGT226 (5 nmol/L) or BEZ235 (50 nmol/L) for 1 h before radiation (4 Gy) and the number of residual foci was determined at 24 hpost-irradiation as previously described [7]. Cells were exposed to PI3K/mTOR inhibitor for up to 24 h post-irradiation. Cells were also treated separately with the BEZ235 (50 nmol/L) and radiation (4 Gy), as above, and a time-course analysis of residual γH2AX foci was performed at 6, 24 and 48 h post-irradiation.

The number of residual DNA damage foci was also measured in HUVEC at 24 h post-irradiation (4 Gy). HUVEC were pretreated with BEZ235 (50 nmol/L) for 1 h before irradiation. Following irradiation, medium was replaced by basal medium containing 1.5% FCS and 10 ng/ml VEGF.

### Cell cycle assay

FaDu, SQ20B and HUVEC cells were plated into T25 tissue culture flasks (Falcon) and incubated overnight to allow cells to reach mid-log phase. Tumor cells were treated with BEZ235 (50 nmol/L) for 1 h before irradiation and medium was replaced 17 h post-irradiation. HUVEC cells were plated in growth-factor depleted medium overnight. Cells were treated with BEZ235 (50 nmol/L) 1 h before irradiation with a single dose of 4 Gy. Following irradiation, HUVEC medium was replaced by basal medium containing 1.5% FCS and a constant concentration of VEGF (10 ng/ml). All cells were trypsinized using 0.5% Trypsin/EDTA (Life Technologies-Bethesda Research Laboratories) and centrifuged at 1200 rpm. Thereafter, they were washed with PBS, resuspended in 1 mL ice cold 70% ethanol and centrifuged again at 1,200 rpm for 10 min. Following this, they were incubated with a mixture of 200 μg/mL RNaseA diluted in PBS with 50 μg/mL propidium iodide (PI), for 30 min at room temperature, in a dark room. Cell cycle was examined 24 h post-irradiation using a Becton Dickinson FACSort machine with the Modfit LT analysis software. Data are representative of three independent experiments.

### Analysis of apoptosis

FaDu, SQ20B and HUVEC cells were plated into T25 tissue culture flasks (Falcon) and incubated overnight to allow cells to reach mid-log phase. Tumor cells were treated with PI3K/mTOR inhibitor for 1 h before irradiation. Medium was replaced 17 h post-irradiation. HUVEC cells were plated in growth-factor depleted medium overnight. Cells were treated with BEZ235 (50 nmol/L) 1 h before irradiation with a single dose of 4 Gy. In tumor cells, fresh medium was replaced 17 h post-irradiation. Similarly to the cell cycle assay, following irradiation, HUVEC medium was replaced by basal medium containing 1.5% FCS and a constant concentration of VEGF (10 ng/ml). Cells were allowed to grow and were finally trypsinized at 24 and 48 h post-irradiation. Apoptosis was analyzed by flow cytometry using FITC Annexin V apoptosis detection kit I (BD Pharmingen) in combination with PI staining, according to the manufacturer's instructions. Analysis of the data was conducted with the FlowJo 7.5 analysis software (Tree Star, Oregon Corporation).

### Capillary tube formation and endothelial cell migration

HUVEC and HDMVC in mid-log phase were plated in growth-factor depleted medium overnight and treated with BEZ235 (50 nmol/L) for 1 h, before irradiation with 4 Gy. Cells were trypsinized immediately after irradiation and plated onto 24-well plates, previously coated with Matrigel (300 μL per well; BD Biosciences), and incubated in basal medium containing 1.5% FCS and a constant concentration of VEGF (10 ng/ml). Once tubules began to form in the control group, cells were stained with calcein (BD Biosciences, Germany), according to the manufacturer's instructions. Three randomly selected digital microphotographs (magnification × 10 for HUVEC and x4 for HDMVC) were obtained from each well. The length of capillary-like tubular structures was measured with the ImageJ software and was normalized to the control group. Experiments were performed twice in quadruplicates.

For the migration assay, cells were trypsinized immediately after irradiation and plated onto the top chamber of 24-well plates (60,000 HUVEC and 30,000 HDMVC/well) with 8 μm matrigel-coated inserts (BD Biosciences). Basal medium (500 μL) containing 1.5% FCS and a constant concentration of VEGF (10 ng/ml) was added to the lower compartment, and cells were incubated for 18 h and allowed to migrate towards the VEGF-containing medium, according to the manufacturer's instructions. Cells were finally scraped off at the upper side of the membrane with a cotton swab and migrated cells were stained with calcein fluorescent dye (BD Biosciences). Three randomly selected digital microphotographs were obtained from each well. The number of migrated endothelial cells (lower side of the membrane) per field (magnification × 10) was counted by microscopy. The results represent the mean number of migrated cells, normalized to the control group, as calculated from 3 random fields in quadruplicates.

### Statistical analyses

The values were expressed as means ± SD. The significance of differences between the means was measured by two-tailed *t*-test or one-way ANOVA using the GraphPad Prism program version 4.0 (GraphPad Software, USA). A value *p *< 0.05 was considered statistically significant.

## Results

### BGT226 and BEZ235 inhibit PI3K and mTOR activity and reduce AKT and S-6 phosphorylation

**Figure 1:**
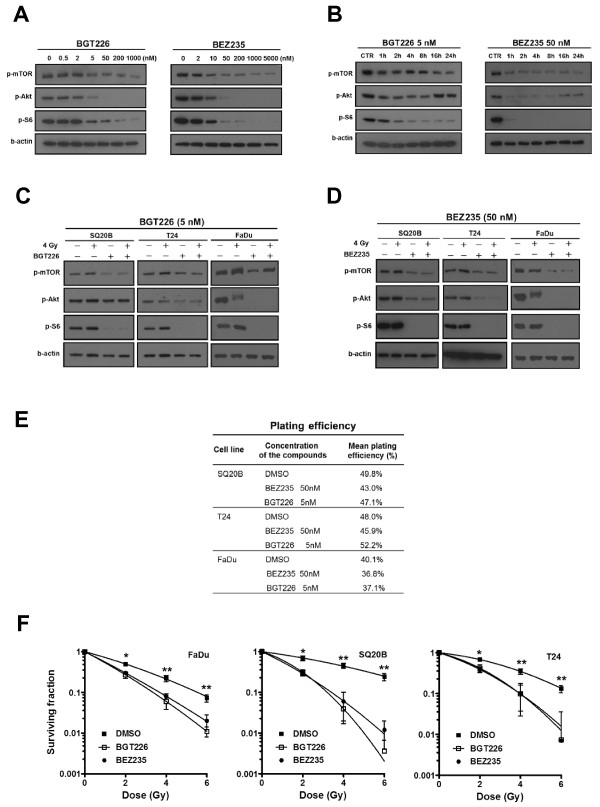
**BGT226 and BEZ235 attenuate oncogenic signaling and reduce clonogenic survival after radiation**. SQ20B cells were exposed to the indicated drugs for 1 h and evaluated by Western blotting for phosphorylation of mTOR (Ser2448), Akt (Ser-473) and S6 protein (ser-235/236). β-actin was used as a loading control. ***A***, Dose response of the PI3K/mTOR pathway in SQ20B cells after 1 h exposure to the indicated drug. ***B***, Time-course of PI3K/mTOR inhibition to indicated drugs at maximal effective doses. ***C-D***, Response of PI3K/mTOR pathway 1 h after 4 Gy. Cells were treated with 5 nmol/L BGT226 (C) or 50 nmol/L BEZ235 (D) for 1 h before and 1 h after irradiation. ***E***, Plating efficiency of SQ20B,T24 and FaDu cells after exposure to indicated drugs for 1 h before up to 17 h post-irradiation (n = 3). ***F***, Clonogenic survival of indicated cell lines after 18 h treatment with BEZ235 (50 nmol/L) and BGT226 (5 nmol/L), as described in "Methods" (n = 3). P < 0.05; **, P < 0.01 over DMSO-treated control.

We initially aimed to confirm inhibition of PI3K and mTOR by these novel compounds and to establish their minimum inhibitory concentrations. To this end, we analysed the phosphorylation of PI3K pathway downstream targets by Western blotting after treatment of SQ20B cells with BGT226 and BEZ235 in increasing concentrations (Figure 1A). BGT226 and BEZ235 were able to inhibit phosphorylation of Ser473 Akt, Ser2448 mTOR, and Ser240/244 S6 in SQ20B cells at concentrations of 5 nmol/L and 50 nmol/L, respectively (Figure 1A). BEZ235 (50 nmol/L) inhibited phosphorylation of all three targets within 1 h of exposure. Inhibition persisted for at least 24 h. Inhibition of pAKT by BGT226 (5 nmol/L) was relieved after 16 h (Figure 1B). Signalling inhibition occurred in irradiated cells as well (Figure 1C and 1D).

### The dual PI3K/mTOR inhibitors reduce radiation survival of tumor cells with EGFR overexpression or Ras mutation

SQ20B and FaDu are derived from head and neck cancers with overexpression of EGFR. T24 is a bladder cancer cell line with mutated H-Ras. We conducted experiments in order to assess the optimal drug incubation time for colony forming assays with BEZ235 (50 nmol/L) and BGT226 (5 nmol/L) in SQ20B,T24 and FaDu cells in the absence of radiation. Exposure of cells to the drugs for 18 h did not alter plating efficiency (PE) significantly (Figure 1E). Therefore, for subsequent clonogenic assays, cells were pretreated with either compound for 1 h before irradiation and total incubation time was limited to 18 h. BGT226 and BEZ235 treatment for 18 h resulted in significant reduction in clonogenic survival after irradiation in all three cell lines (Figure 1F). To quantify the effect, the radiation dose required to reduce the surviving fraction to 10% was calculated (dose modifying factor 10%; DMF10). The ratio of DMF10 in control cells to BGT226-treated cells was calculated to be 2.6 for SQ20B, 1.6 for FaDu and 1.7 for T24. In BEZ235-treated cells, the DMF10 was 2.5 for SQ20B, 1.5 for FaDu and 1.7 for T24. Thus, there is significant radiosensitisation of these three cell lines by these inhibitors.

To understand the mechanisms of radiosensitisation, we investigated BGT226- and BEZ235-induced enhancement of radiation response in the post-irradiation setting. BGT226 (5 nmol/L) or BEZ235 (50 nmol/L) were added to the culture medium of SQ20 and T24 cells immediately or 6 h after exposure to radiation, for a total exposure time of 18 h. Treatment with drug immediately after irradiation was similar to giving the drug before (Additional file 1: Figure S1A) but if given 6 h after exposure, no radiosensitizing effect was observed (Additional file 1: Figure S1B). The latter indicates that blockade of the PI3K/mTOR pathway early before or after irradiation is necessary for sensitizing tumor cells to radiation damage.

### BEZ235 radiosensitises tumor cells under hypoxic conditions

Because hypoxic cells can be up to three fold more radioresistant than normoxic cells [1], we asked whether the radiosensitising effect of BEZ235 can still be seen under hypoxic conditions (Additional file 2: Figure S2A-B). Tumor cells were treated with one of the inhibitors, BEZ235 (50 nmol/L) for 1 h before up to 17 h after irradiation under hypoxic conditions (0.5% oxygen). Treatment with BEZ235 in the absence of irradiation did not result in significant toxicity in hypoxia (data not shown). Addition of BEZ235 reduced post-irradiation survival significantly for all three cell lines in hypoxia (Additional file 2: Figure S2A). All cell lines showed increased radioresistance under hypoxic conditions, as compared to normoxia, confirming the hypoxic effect in our experimental settings (Additional file 2: Figure S2B). These results show that PI3K/mTOR inhibition can radiosensitise tumor cells in normoxic as well as hypoxic conditions.

### BEZ235 induces apoptosis in SQ20B cells and increases necrosis

**Figure 2:**
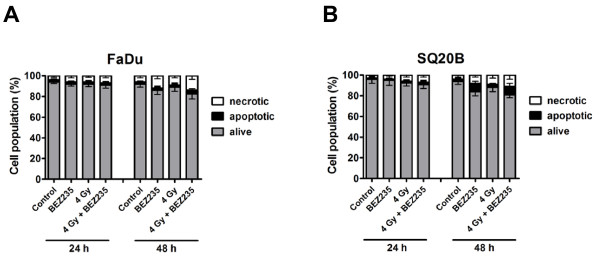
**Evaluation of apoptosis in irradiated tumor cells treated**. ***A-B***, Cells were treated with BEZ235 (50 nmol/L). 4 Gy was given after 1 h of drug treatment, with the drug maintained for an additional of 17 h. Apoptosis was assessed by flow cytometry for Annexin V combined with propidium iodide, at 24 and 48 h post-irradiation (n = 2).

We analysed apoptosis in FaDu and SQ20B cells upon administration of BEZ235 (50 nmol/L), in combination with irradiation (4 Gy) (Figure 2A-B). We did not observe any increase in apoptosis in FaDu cells after treatment with BEZ235 alone at either time point while necrosis was increased, especially at 48 h post-irradiation. In contrast, BEZ235 increased both apoptosis and necrosis at 48 h after irradiation in SQ20B cells. Radiation alone enhanced necrosis at 48 h post-irradiation in FaDu and SQ20B cells. (Figure 2A-B). The addition of BEZ235 to radiation did not increase apoptosis in either cell line. Only a slight increase in necrosis was observed at 48 h post-irradiation in both cell lines.

### Radiosensitisation induced by the dual PI3K/mTOR inhibitors is accompanied by persistence of γH2AX foci and cell cycle arrest

**Figure 3:**
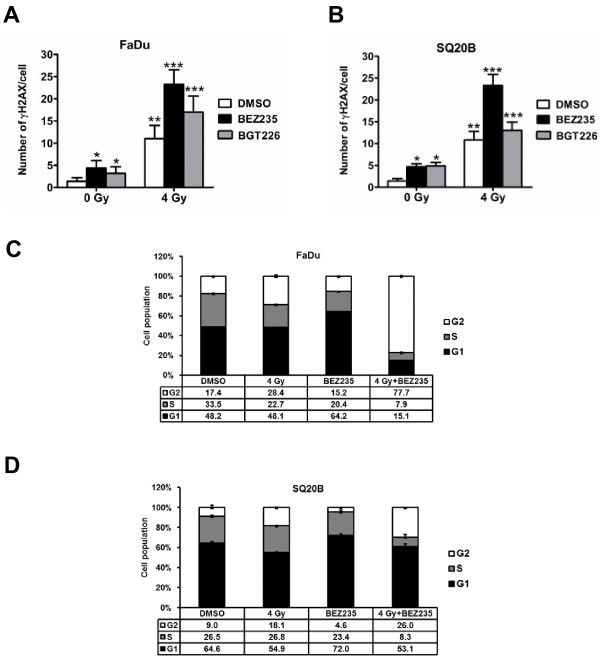
**Evaluation of residual DNA damage and cell cycle distribution in irradiated tumor cells**. ***A-B***, γH2AX foci at 24 h post-irradiation. Indicated cell lines were exposed to 5 nmol/L BGT226 or 50 nmol/L BEZ235 for 1 h followed by irradiation with 4 Gy. Drugs were left until staining was performed. Residual γH2AX foci were counted 24 h later. ***C-D***, Cells were treated as in (A) and cell cycle distribution was determined by flow cytometry after exposure to propidium iodide 24 h post-irradiation (n = 3). *, *P *< 0.05; **, *P *< 0.01; ***, *P *< 0.001 over DMSO-treated control.

To gain insight into the molecular mechanisms of radiosensitization of both compounds, we investigated the effect of these drugs on the DNA damage response by measuring the number of γH2AX foci at different time points post-irradiation (4 Gy). A higher number of residual γH2AX foci was detected after treatment with BGT226 (5 nmol/L) and BEZ235 (50 nmol/L) as compared with radiation alone, at 24 h post-irradiation (Figure 3A-B). We confirmed the higher number of foci after treatment of cells with BEZ235 at different time points post-irradiation in tumor cells (Additional file 3: Figure S3A-B). Although the number of foci decreases more rapidly in FaDu after radiation alone, the trend at 12, 24 and 48 h is similar for both FaDu and SQ20B cells and reveals approximately twice as many foci in the combination group, as compared to radiation alone.

We also investigated the impact of PI3K/mTOR inhibition on cell cycle distribution. Treatment with BEZ235 (50 nmol/L) for 1 h before irradiation up to 17 h after led to an increased percentage of cells in G1 phase while S decreased, indicating a G1 block. Irradiation of FaDu cells led to a G2 block that was substantially increased after treatment with the inhibitor (Figure 3C). Similar effects were obtained from SQ20B cells even though the increase in G2 phase delay in the combination group was less dramatic (Figure 3D). The profound G2 block observed in the combination group underlines the radiosensitizing potential of these drugs.

### BEZ235 blocks PI3K/mTOR signaling and sensitizes endothelial cells to irradiation

**Figure 4:**
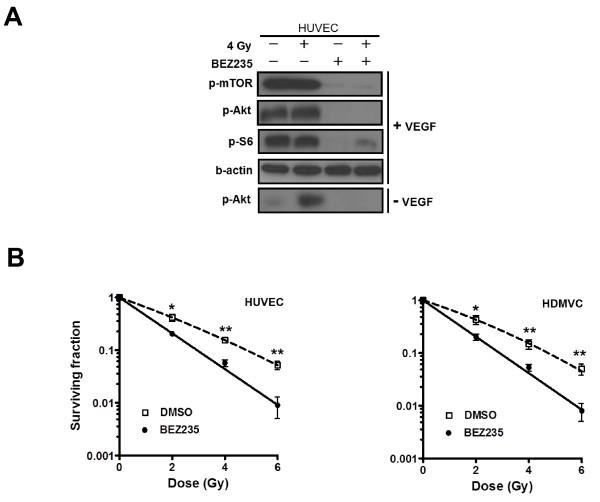
**PI3K/mTOR inhibition affects downstream signaling and clonogenic survival in irradiated vascular endothelial cells**. ***A***, HUVEC were starved overnight and were treated with 50 nmol/L BEZ235 for 1 h before irradiation (4 Gy). Following irradiation, medium was replaced by either basal medium containing 1.5% FCS and 10 ng/ml VEGF (+VEGF) or growth factor-depleted medium (-VEGF). Cell lysates were collected 1 h post-irradiation and subjected to Western blot analysis. ***B***, Clonogenic survival of HUVEC and HDMVC treated with BEZ235 (50 nmol/L) for 1 h before irradiation up to 1 h post-irradiation (n = 3). *, *P *< 0.05; **, *P *< 0.01 over DMSO-treated control.

Next we wanted to investigate the effect the dual PI3K/mTOR inhibitors in endothelial cells. To this end, we determined the effect of irradiation and VEGF on the PI3K signalling pathway in HUVEC using BEZ235 (50 nmol/L). In endothelial (HUVEC) cells, Akt was phosphorylated 1 h after irradiation (4 Gy) or exposure to VEGF-containing medium (10 ng/mL) (Figure 4A; +VEGF). HUVEC exposed to growth factor-depleted medium did not show Akt phosphorylation (Figure 4A; -VEGF). Pre-treatment of HUVEC with BEZ235 led to complete abrogation of PI3K/Akt/mTOR signalling, in irradiated and unirradiated HUVEC (Figure 4A). Treatment of HUVEC cells with BEZ235 (50 nmol/L) for 1 h before up to 1 h after irradiation significantly reduced clonogenic survival in HUVEC (Figure 4B). A similar decrease in clonogenicity was observed in HDMVC, cells that more closely resemble tumor microvascular cells (Figure 4B).

### BEZ235 increases DNA damage and necrosis in irradiated endothelial cells

**Figure 5:**
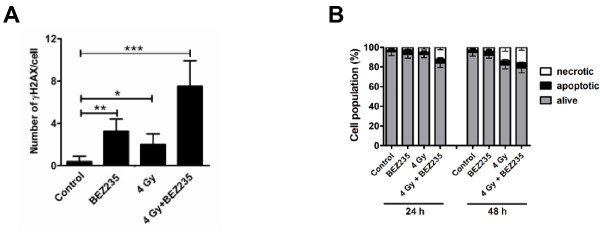
**PI3K/mTOR inhibition enhances DNA damage in irradiated HUVEC**. ***A***, apoptosis assessment in HUVEC treated with BEZ235 and/or radiation. Previously starved HUVEC were treated with BEZ235 (50 nmol/L) for 1 h before irradiation. The medium was removed immediately following irradiation and was replaced by basal medium containing 1.5% FCS and a constant concentration of VEGF (10 ng/ml).. Annexin V-propidium iodide flow cytometry was used to identify cells in apoptosis or necrosis 72 h after irradiation. ***B***, mean γH2AX foci number in HUVEC cells treated with BEZ235 (as above) and radiation at 24 h post-irradiation (n = 3). *, *P *< 0.05; **, *P *< 0.01; ***, *P *< 0.001 over DMSO-treated control.

We analysed DNA damage in irradiated cells pretreated with BEZ235 (50 nmol/L) in response to VEGF (10 ng/mL), as described in Materials and Methods. BEZ235 resulted in enhanced persistence of γH2AX foci 24 h after exposure to 4 Gy irradiation (Figure 5A). In addition, BEZ235 treatment only slightly increased apoptosis and necrosis at 24 and 48 h and enhanced radiation-induced necrosis, especially at 24 h post-irradiation (Figure 5B). Radiation alone increased necrosis 48 h after radiation. (Figure 5B).

### BEZ235 attenuates tube formation and migration in irradiated endothelial cells

**Figure 6:**
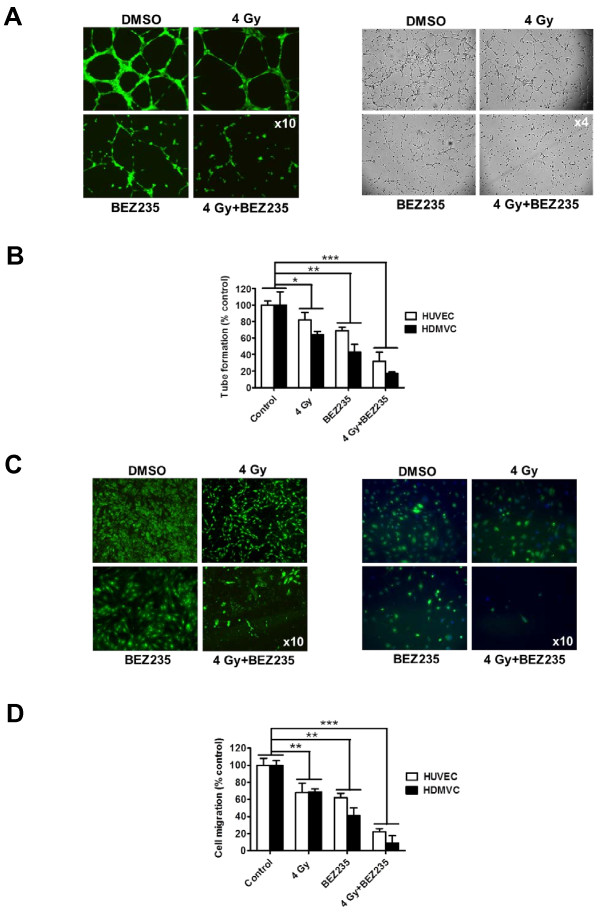
**PI3K/mTOR inhibition attenuates tubule formation and migration by irradiated HUVEC and HDMVC**. Cells were exposed to BEZ235 (50 nmol/L) for 1 h, before irradiation with 4 Gy. Cells were trypsinized immediately after irradiation and were incubated in basal medium containing 1.5% FCS and a constant concentration of VEGF (10 ng/ml). ***A***, representative images of capillary tubule formation in HUVEC (left) and HDMVC (right) in response to BEZ235 (50 nmol/L). ***B***, quantification of tubule formation as measured by the length of capillary-like tubular structures and normalized to the control group (4 power fields per sample). ***C***, representative images of HUVEC (left) and HDMVC (right) migration 18 h post-irradiation. ***D***, endothelial cell migration of indicated cells (treated as above). The number of migrated cells per power field (10X) was counted (12 fields per group) and normalized to the control group (n = 3). *, *P *< 0.05; **, *P *< 0.01; ***, *P *< 0.001 over DMSO-treated control.

To determine whether Akt/mTOR inhibition affects the formation of vascular networks by endothelial cells, a tube formation assay was performed as described in Materials and Methods. BEZ235 (50 nmol/L) or irradiation alone (4 Gy) decreased tube formation in both HUVEC and HDMVC and resulted in shorter tubular structures with fewer interconnection branching points (Figure 6A-B). The combination of BEZ235 with irradiation further potentiated the reduction (Figure 6A-B).

For the migration assay, cells were treated in a similar way as in the tube formation assay and were allowed to migrate to the lower compartment of a transwell chamber. BEZ235 (50 nmol/L) and irradiation (4 Gy) significantly reduced migration of HUVEC and HDMVC (Figure 6C-D). Addition of BEZ235 to radiation revealed inhibition of endothelial cells migration (Figure 6C-D). Therefore, dual PI3K/mTOR inhibition can enhance the antivascular effect of radiation in culture.

## Discussion

Our past work and that of others point to increased PI3K/Akt/mTOR signalling as a mediator of enhanced tumor survival after radiation-induced DNA damage [3,7,9,24]. Deregulation of mTOR signalling has also been implicated in response to radiation [14]. Rapalogs have antiproliferative effects in vitro but their efficacy in tumors with PI3K/Akt and mTOR activation has been limited. There is extensive crosstalk between the two signalling networks [14,17]. mTOR can affect PI3K/Akt signalling through the S6K-IRS1 feedback loop and induce Akt phosphorylation by mTORC2 [14,25]. Because rapalogs inhibit only the mTORC1 complex, paradoxical activation of Akt can limit their therapeutic potential. Here we have shown that PI3K/mTOR dual inhibitors effectively block downstream targets and result in radiation sensitization in tumor cell lines and in endothelial cells. Interestingly, PI3K/mTOR inhibition resulted in decreased clonogenicity in cells radiated in hypoxia. These data indicate that dual PI3K/mTOR inhibition might prevent PI3K pathway reactivation and further enhance radiation-induced cell killing.

Several preclinical studies have found promising activity for the dual PI3K/mTOR inhibitor BEZ235 against various tumors [18,19] especially those with mutations in PI3K. In the present study, dual inhibitors led to radiosensitization of tumor cells and of endothelium. The efficacy of these compounds should apply to tumor cells with a wide spectrum of oncogenic lesions because the Ras/EGFR/PI3K/mTOR pathway is activated in many types of cancer. Both BGT226 and BEZ235 enhanced the radiosensitivity of SQ20B cells and T24 cells when added before or immediately after radiation but not after 6 h. These findings may support scheduling strategies for future clinical trials testing the radiosensitising potential of these compounds.

To determine whether radiosensitisation was associated with inhibitor-mediated cell cycle redistribution, we analysed cycle distribution in cells pretreated with one of the dual inhibitors, BEZ235. Treatment of FaDu and SQ20B cells with BEZ235 alone resulted in growth arrest in the G_1 _phase.. This is similar to the observation reported in several studies investigating BEZ235 and other PI3K inhibitors [18,26-28]. Importantly, when cells were irradiated after BEZ235 pretreatment, the percentage of SQ20B and FaDu cells in G2 phase was increased by approximately 3-fold and 4.5-fold, respectively. This finding concurs with our previous report on PI3K inhibitor, PI-103 where a ~2-fold increase in G2 phase population arrest was recorded [7]. Notably, rapalogs are known to induce a G_2 _block when combined with irradiation [14].

We also investigated the effect of dual PI3K/mTOR inhibition in apoptosis. BEZ235 increased necrosis but not apoptosis in FaDu cells. In contrast, BEZ235 enhanced both apoptosis and necrosis in SQ20B cells. In the combination group, there was no increased apoptosis in either cell line and only a slight increase in necrosis was observed at 48 h post-irradiation. Previous studies have demonstrated increased apoptosis after treatment with BEZ235 in some tumor cell lines and lack of apoptosis induction in others. For instance there was no apoptosis induction in glioma or melanoma cell lines [26,27]. There is however in lung cancer, sarcoma and leukemia [28-30].

Hypoxic cells are 2 to 3-fold more resistant than oxic cells to radiation and tumor hypoxia is associated with treatment failure following radical radiotherapy [1]. We were therefore interested to investigate the efficacy of BEZ235 in the context of hypoxia. As expected, hypoxia resulted in increased radioresistance of FaDu, SQ20B and T24 cells. Importantly, PI3K/mTOR inhibition by BEZ235 led to significant sensitization of hypoxic cells to radiation and therefore this drug can be an attractive adjunct for radiotherapy.

BEZ235 and BGT226 enhanced persistence of residual γH2AX foci after irradiation. γH2AX foci were also moderately increased in cells treated with BEZ235 alone, which could be attributed to the potentially toxic effect of the compounds, leading to enhanced DNA damage even in the unirradiated cells. Selective inhibition of the PI3K pathway using siRNA leads to significant radiosensitization of tumor cells. Therefore, the radiosensitizing effect of PI3K/mTOR inhibitors cannot be wholly attributed to inhibition of other targets ("off-target" effect). Previous evidence has demonstrated that inhibition of the PI3K pathway can affect formation of γH2AX foci, even in the absence of radiation [7]. These indicate that PI3K/mTOR plays a role in DNA repair after the initial injury. Our results are in accordance to the work of Konstantinidou et al. [29]. Similar findings have been also been described before for different PI3K inhibitors [7,31].

The PI3K/Akt/mTOR intercept node is involved in endothelial signaling response to upstream effectors such as VEGF [22]. Chronic Akt activation in endothelial cells recapitulated the salient features of tumor vasculature [32]. In VEGF-stimulated porcine aortic endothelial cells and HUVEC, VEGFR2 recruited the p110/p85 complex and increased their proliferation [33]. PI3K/Akt/mTOR activation can occur upon exposure to radiation in endothelial cells [22]. Overexpression of Akt in endothelial cells resulted in abnormal vascular remodeling with embryonic lethality [34]. Here BEZ235 blocked VEGF- and irradiation-induced activation of Akt phosphorylation and significantly enhanced cell death in vascular and microvascular endothelial cells. Furthermore, BEZ235 reduced VEGF-mediated migration and tube formation and enhanced the antivascular effect of radiation in endothelial cells. We observed a slight increase in apoptosis and necrosis in BEZ235-treated endothelial cells. BEZ235 increased radiation-induced necrosis, especially at 24 h post-irradiation. Our findings are in accordance with previous reports showing that PI3K and/or mTOR blockade can exert an antivascular activity [15,22,35]. The mTOR inhibitor rapamycin decreased VEGF-mediated growth of endothelial cells and activation of Akt/mTOR signaling after irradiation and enhanced the antivascular efficacy of radiotherapy [15,16,22]. The fact that dual inhibition of PI3K/mTOR pathway can increase the antivascular effect of radiation in endothelial cells is an important finding. First, PI3K/mTOR inhibition by BEZ235 alone can result in alterations in tumor blood vessel morphology andfunctionality but this appears to be a dose-dependent effect and can affect the efficacy of radiotherapy significantly, as recently demonstrated by our group [36]. Secondly, the enhanced radiosensitization of endothelial cells conferred by BEZ235 would imply that dual PI3K/mTOR inhibition could, in theory, enhance normal tissue damage and therefore special caution is required before proceeding with clinical studies using these agents in combination with radiation.

## Conclusions

In summary, we demonstrated that PI3K/mTOR dual inhibitors are effective radiosensitisers in tumor cells with EGFR overexpression or oncogenic Ras mutation. BEZ235 sensitized tumor cells to radiation under both normoxic and hypoxic conditions. The antivascular activity reported represents a clear advance in understanding the properties of dual PI3K/mTOR inhibition. Altogether, our data indicate that direct inhibition of PI3K/mTOR activity may be of benefit when combined with radiotherapy.

## Abbreviations

PI3K: Phosphatidylinositol 3-kinase; mTOR: Mammalian target of rapamycin; EGFR: Epidermal growth factor receptor; PTEN: Phosphatase and tensin homologue deleted in chromosome 10; VEGF: Vascular endothelial growth factor; HUVEC: Human umbilical vein endothelial cells; HDMVC: Human dermal microvascular cells; DMF10: Dose modifying factor 10%; PE: Plating efficiency; PI: Propidium iodide.

## Competing interests

S-M Maira and W. Hackl are Novartis Pharma employees and shareholders. The other authors disclosed no potential competing interest.

## Authors' contributions

EF and MY are co-first authors. EF and MY conducted the majority of the experimental procedures. RP and GH participated in the western blots and DNA damage foci analyses. WH and SMM provided the drugs and scientific advice. EB, WGM and RJM conceived the study, and participated in its design and coordination. EM, MY and RJM drafted the manuscript. All authors read and approved the manuscript.

## Supplementary Material

Additional file 1 **Figure S1 The effect of timing of BEZ235 and BGT226 administration on tumor cell radiosensitivity**. Clonogenic survival assays of SQ20B and T24 cells treated as indicated. BEZ235 (50 nmol/L) and BGT226 (5 nmol/L) were added ***(A) ***immediately or ***(B) ***6 h following irradiation (n = 3). *, *P *< 0.05; **, *P *< 0.01. Click here for file

Additional file 2 **Figure S2 BEZ235 radiosensitises tumor cells under hypoxic conditions**. ***A***, clonogenic survival curves of cells treated with 50 nmol/L BEZ235 and irradiation under hypoxic conditions. Cells were incubated in hypoxia (0.5% O_2_) for 6 h. BEZ235 was then added at 1 h prior to irradiation and left for 17 h upon and the medium was replaced. Cells were transferred to normoxia at 1 h post-irradiation. ***B***, clonogenic survival of cells after irradiation with 6 Gy and treatment with 50 nmol/L BEZ235 in oxic and hypoxic (0.5% O_2_) conditions, as described above (A) and in Figure 1. *, *P *< 0.05; **, *P *< 0.01; *, *P *< 0.001 over DMSO-treated control. Click here for file

Additional file 3 **Figure S3 Time-course of γH2AX foci in irradiated tumor cells treatedwith BEZ235**. FaDu and SQ20B cells were exposed to 50 nmol/L BEZ235 for 1 h followed by irradiation with 4 Gy. Drugs were left up to a maximum of 24 h. Residual γH2AX foci were counted at the indicated time points. *, *P *< 0.05; **, *P *< 0.01; ***, *P *< 0.001 over DMSO-treated control. Click here for file
