# Supplementary figures and images for: NVP-BEZ235 and NVP-BGT226, dual phosphatidylinositol 3-kinase/mammalian target of rapamycin inhibitors, enhance tumor and endothelial cell radiosensitivity
Source: Radiat Oncol. 2012 Mar 27;7:48. doi: 10.1186/1748-717X-7-48 (PMC3348043; doi:10.1186/1748-717X-7-48)

## Slide 1
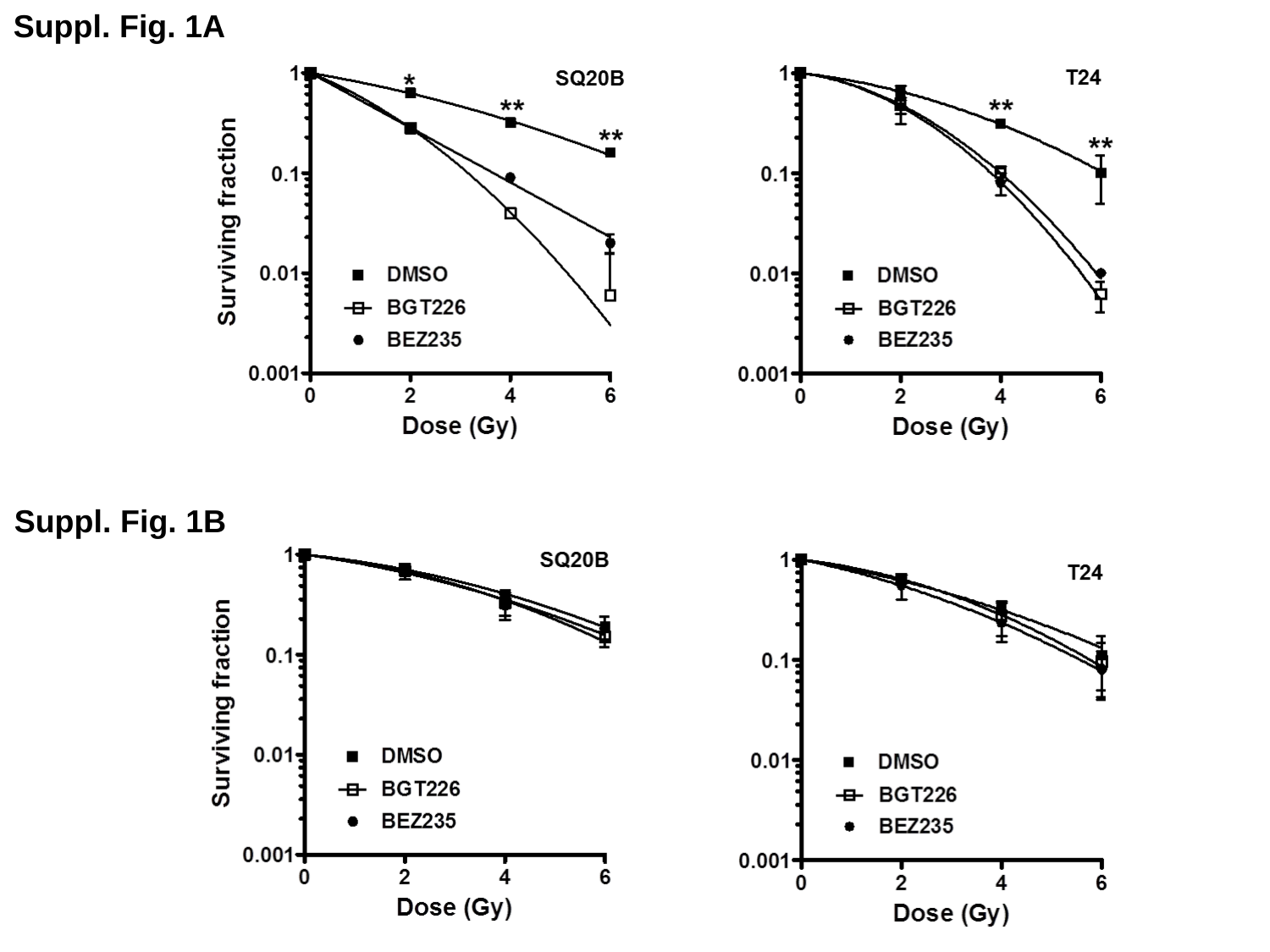

Suppl. Fig. 1A
Suppl. Fig. 1B

Supplement: Additional file 1 — Figure S1 The effect of timing of BEZ235 and BGT226 administration on tumor cell radiosensitivity. Clonogenic survival assays of SQ20B and T24 cells treated as indicated. BEZ235 (50 nmol/L) and BGT226 (5 nmol/L) were added (A) immediately or (B) 6 h following irradiation (n = 3). *, P < 0.05; **, P < 0.01. [file 1748-717X-7-48-S1.PPT]

## Slide 1
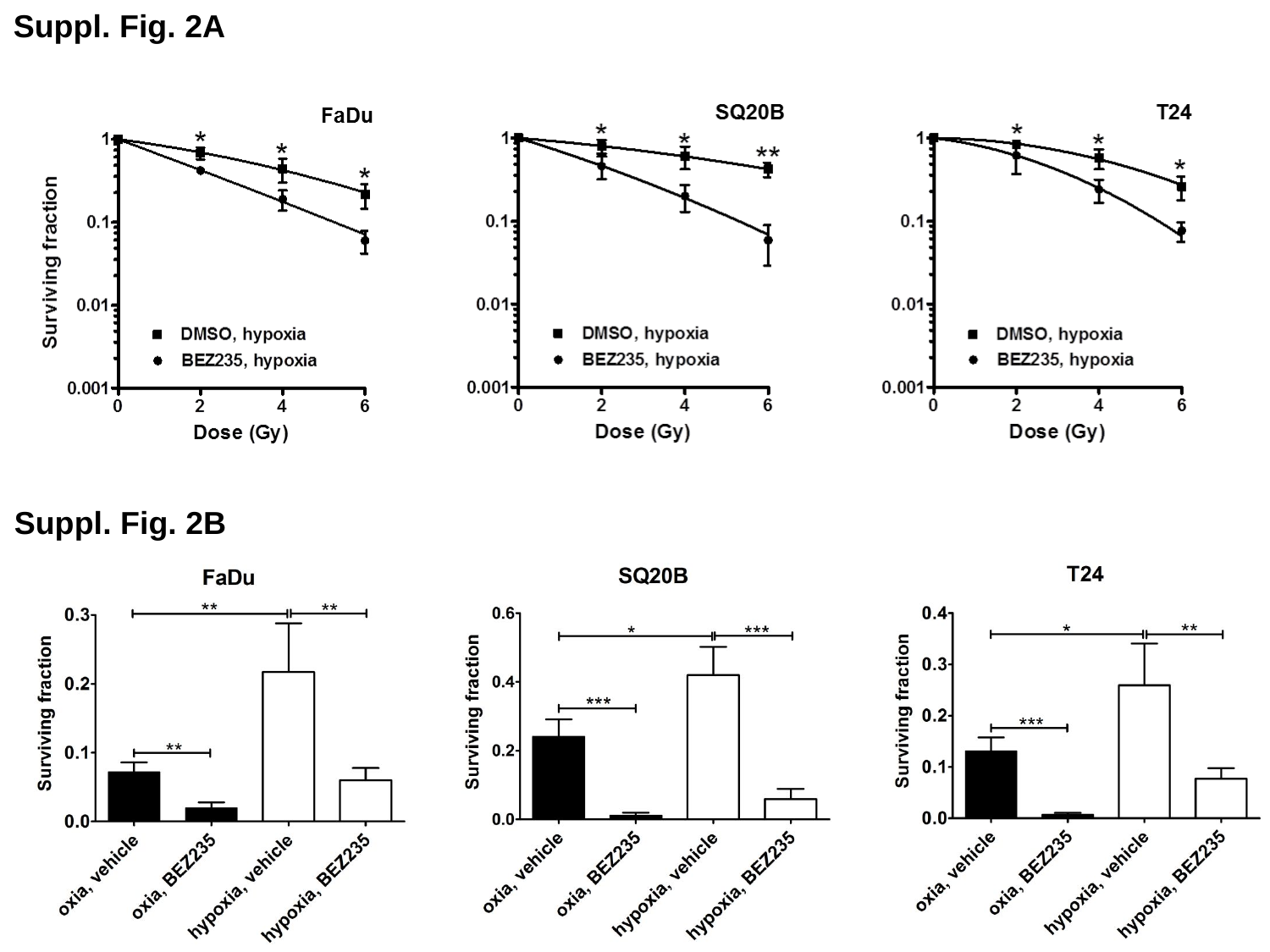

Suppl. Fig. 2A
Suppl. Fig. 2B

Supplement: Additional file 2 — Figure S2 BEZ235 radiosensitises tumor cells under hypoxic conditions. A, clonogenic survival curves of cells treated with 50 nmol/L BEZ235 and irradiation under hypoxic conditions. Cells were incubated in hypoxia (0.5% O2) for 6 h. BEZ235 was then added at 1 h prior to irradiation and left for 17 h upon and the medium was replaced. Cells were transferred to normoxia at 1 h post-irradiation. B, clonogenic survival of cells after irradiation with 6 Gy and treatment with 50 nmol/L BEZ235 in oxic and hypoxic (0.5% O2) conditions, as described above (A) and in Figure 1. *, P < 0.05; **, P < 0.01; *, P < 0.001 over DMSO-treated control. [file 1748-717X-7-48-S2.PPT]

## Slide 1
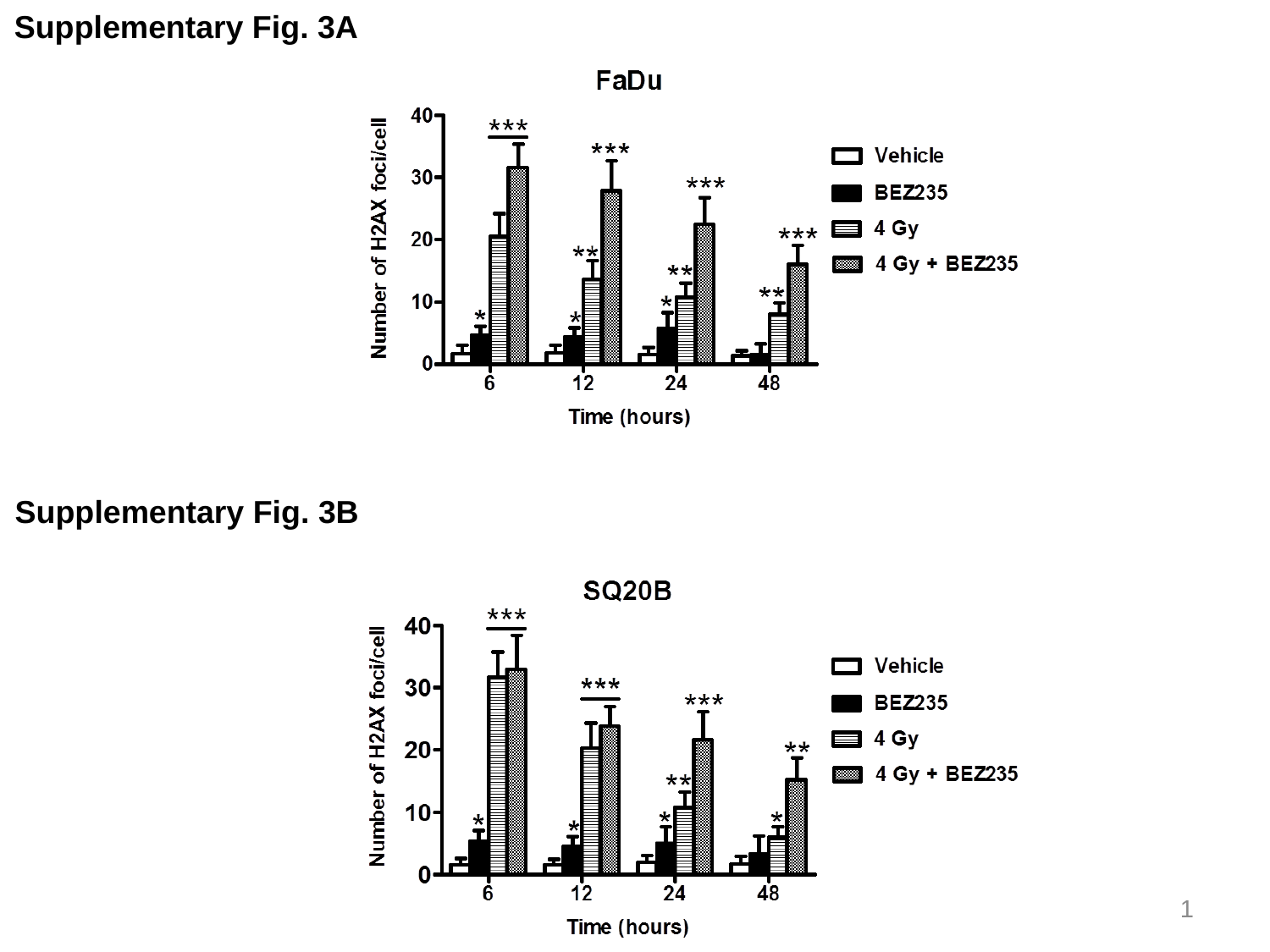

Supplementary Fig. 3A
Supplementary Fig. 3B
1

Supplement: Additional file 3 — Figure S3 Time-course of γH2AX foci in irradiated tumor cells treatedwith BEZ235. FaDu and SQ20B cells were exposed to 50 nmol/L BEZ235 for 1 h followed by irradiation with 4 Gy. Drugs were left up to a maximum of 24 h. Residual γH2AX foci were counted at the indicated time points. *, P < 0.05; **, P < 0.01; ***, P < 0.001 over DMSO-treated control. [file 1748-717X-7-48-S3.PPTX]
